# The Editor's Letter-Box

**Published:** 1888-09-29

**Authors:** Lucy Robson

**Affiliations:** S. Bede's House, Fairfax Road, Swiss Cottage.


### EDITOR'S LETTER-BOX.

[Our Correspondents are reminded that prolixity is a great bar to publication, and that brevity of style and conciseness of statement greatly facilitate early insertion.]

As a constant reader of THE HOSPITAL, I see from time to time paragraphs calling attention to a want felt by many for a supply of nurses on reduced terms and for short periods. For some years I have been sending out nurses by the hour, day or night, as I find from experience that a great number, including bachelors and others, can ill afford to pay the full weekly fee, and yet require skilful nursing in lodgings when in a sick and helpless condition, separated from friends and home. As the nurses from this institution receive their own earnings, they are more in a position to give their services for such a purpose than institutions who pay their nurses a salary. I may also mention another admirable institute which has done such good service amongst people of reduced circumstances, namely, "The Metropolitan Nurses," Edgware Road.

LUCY ROBSON.

S. Bede's House, Fairfax Road, Swiss Cottage.

